# Lived experiences of children and adolescents with obsessive–compulsive disorder: interpretative phenomenological analysis

**DOI:** 10.1186/s13034-022-00478-7

**Published:** 2022-06-16

**Authors:** Lakshmi Sravanti, John Vijay Sagar Kommu, Satish Chandra Girimaji, Shekhar Seshadri

**Affiliations:** 1 Department of Child and Adolescent Psychiatry, National Institute of Mental Health and Neurosciences (NIMHANS), Bengaluru, India; 2 F-102, Concorde Manhattans, Electronic City Phase -1, Bengaluru, 560100 India

**Keywords:** Lived experiences, Children, Adolescents, Obsessive–compulsive disorder

## Abstract

**Background:**

Childhood obsessive–compulsive disorder (OCD) is distinct from OCD in adults. It can be severely disabling and there is little qualitative research on OCD in children. The present study aims to explore the subjective experiences of diagnosis, treatment processes and meaning of recovery in children and adolescents suffering from OCD and provide a conceptual model of the illness.

**Methods:**

It is a qualitative study of ten children and adolescents selected by purposive sampling. MINI KID 6.0, Children’s Yale-Brown Obsessive–Compulsive Scale and Clinical Global Impression-Severity Scale were administered at the time of recruitment of subjects into the study. Interviews were conducted using an in-depth semi-structured interview guide and audio-recorded. The transcribed interviews were analyzed using Interpretative Phenomenological Analysis (IPA). The study sought to explore participants’ sense-making of their world, their thoughts, feelings and perceptions through interpretative enquiry. The findings were confirmed by a process called investigator triangulation, member check and peer validation.

**Results:**

IPA yielded five major themes—‘illness perception changes over time’, ‘disclosure on a spectrum’, ‘cascading effects of OCD’, ‘treatment infuses hope and helps’, and ‘navigating through OCD’. A summary of these themes and their subthemes is presented as a conceptual model. The essence of this model is to show the inter-relationship between themes and provide a comprehensive understanding of the phenomenon of OCD.

**Conclusions:**

To the best of our knowledge, this is the first study to explore lived experiences of children and adolescents with OCD using interpretative phenomenological analysis (IPA). It was noted that perception of illness and treatment processes evolves over time, and recovery is viewed as a process. Future qualitative research can be carried out with a focus on ‘therapist-related barriers’ or ‘student–teacher dyads’ that can inform clinical practice and school policies respectively.

*Trial registration* NIMH/DO/IEC (BEH. Sc. DIV)/2018, l1 April 2018.

## Key message

- Delay in recognition of symptoms contributes to significant distress to the individual and family in cases where help is sought.
- There is a need to improve awareness so as to identify the problems early. This will improve help-seeking behaviour and aid in reducing peer victimization.
- Therapist-related barriers contribute to delays in making a diagnosis.
- Treatment is likely to positively impact other spheres of the child’s life such as academics and interpersonal relationships and result in more long-term benefits.
- Bullying in school-context is a common occurrence.

## Introduction

Pierre Janet described pediatric obsessive–compulsive disorder (OCD) for the first time in a 5-year-old boy [1]. OCD with onset in childhood appears to be a distinct subtype with a unique clinical, etiological and epidemiological profile [2, 3]. It has a higher prevalence of comorbid ADHD, anxiety and tic disorders [4], higher familial and genetic loading [5], and higher persistence rates [6] as compared to the adult-onset OCD. When OCD has its onset in childhood, it interferes with normal development and poses a higher likelihood of anxiety disorders in adulthood [7]. It was found that children usually minimize obsessive–compulsive symptoms when compared to parental reports [8]. Treatment delay is common and is associated with poorer outcomes. Diagnosis is generally delayed by three years after the onset of symptoms [9]. Factors leading to delay in recognition and diagnosis are lack of insight, shame associated with symptoms, family accommodation and lack of awareness of the disorder both among patients and clinicians [10].

In addition, there are social issues surrounding OCD in children with classroom implications being at the forefront [11]. Storch et al. reported that more than one-fourth of the sample with OCD faced peer victimization on a regular basis [12]. This condition in children can even be associated with low self-esteem and social ostracization [11]. Bhattacharya and Singh described ‘feeling different from others’ and a loss of an authentic self in youth aged 18–25 years in their thematic content analysis [13]. Brooks interpreted OCD as a traumatic brain disorder that impairs a person’s public and private identities causing significant mental disability [14]. This suggests that an individual’s true self is masked by the face of illness, which may gradually disappear in the due course of time. Therapists often tend to focus on symptom reduction. Symptom reduction is only one aspect of “treatment”. It is imperative to shift focus to enabling children and adolescents to live their lives with dignity by providing holistic care and helping them develop an authentic sense of self.

Subjective experiences or otherwise called lived experiences are often a subject of interest and appealing to study in the field of human psychology and psychiatry. Literature on the subjective experience of severe mental illnesses has laid emphasis on three major aspects viz. the person’s responses and attitudes to his or her illness, the degree of awareness of the illness and the experience of illness as a traumatic event [15]. Interpretative Phenomenological Analysis (IPA) aims to explore lived experience of a phenomenon through the subject’s personal experiences and perception of objects and events. Its hallmark is that the researcher not only gets an insider’s perspective but also plays an active role in interpreting the process and experience [16].

Qualitative data on the phenomenon of OCD in children is limited as compared to the amount of quantitative research done in this field. Most of the findings are from work done in adult participants. Some aspects of pediatric OCD that have been studied are coercive and disruptive behaviours [17], parental adaptation [18], caregiver’s experiences [19], and therapists’ perception of ERP [20]. The study participants in these studies were either the family members [17–19] or therapists [20] and not the children. To the best of our knowledge, there are only two qualitative studies done in adolescents with OCD that have been published. Lenhard et al., studied adolescents’ experiences of internet-delivered CBT [21]. Effectively, there is only one study published that explored the lived experiences of adolescents with OCD [22]. Table 1 summarizes the phenomenon, age group and the number of children studied, methodological approach, and key findings of these two studies.

**Table 1:** Qualitative research done in children and adolescents with OCD

Authors & Place	Phenomenon	Sample	Method	Key findings
1. Keyes, Nolte & Williams, 2018 (UK)	Lived experiences	13–18 years; (n = 10)	Thematic analysis	Four themes: Traumatic and stressful life events; responses to signs of OCD; battle of living with OCD and ambivalent relationship to help; need to address stigma and sense of shame [22]
2. Lenhard et al., 2016 (Sweden)	Experiences of Internet-delivered CBT	12–17 years; (n = 8)	Thematic analysis	Two superordinate themes -autonomy and support. Each with 3 sub-themes (secure self-disclosure, self-efficacy, flexibility & parental support, identification/normalization, clinician support respectively) [21]

Despite the obvious differences in illness presentation in children as compared to adults such as the unique comorbidity profile of pediatric OCD or its varying levels of insight, the treatment strategies are similar in both the age groups. Moreover, given the developmental differences, children may experience this phenomenon differently. To our knowledge, there is only one published study that explored the lived experiences of adolescents aged 13 years to 18 years by using thematic analysis and no study done in younger children. So, clearly, there is a dearth of literature in this area. There is no data on the in-depth analysis of the subjective experiences of children and adolescents with obsessive–compulsive disorder with a focus on illness perception, perception of its impact on functioning and treatment processes and the meaning of recovery. IPA is the method of choice to explore lived experiences. We address this felt need to study the first-hand accounts of children and adolescents suffering from OCD through our study. This study aims to comprehensively analyze the subjective experiences of children and adolescents living with OCD and provide a conceptual model of the lived experiences of the phenomenon of childhood OCD. The findings generated will improve our understanding of their subjective perceptions and help in devising a plan for providing holistic care. The objective is to help clinicians in connecting with these children to gain better insight into their inner world during the process of treatment.

## Methods

### Study design

A qualitative exploratory study using an in-depth, individual, semi-structured interviews was conducted. The interview guide was developed according to the interpretative phenomenological analysis (IPA) guidelines. At the end of each interview, significant observations were noted that were used while interpreting the data to get a better understanding of each subject’s account. The study was conducted in a naturalistic setting of the out-patient and in-patient settings of the department of child and adolescent psychiatry at the National Institute of Mental Health and NeuroSciences (NIMHANS). The study population constituted of children and adolescents diagnosed with obsessive–compulsive disorder selected by purposive sampling.

The primary objective was to explore subjects’ perception of illness, the experience of others’ perceptions, treatment and meaning of recovery. The secondary objective was to identify barriers and facilitators to recovery and design a recovery model. The data pertaining to recovery (barriers, facilitators and recovery model) is a subject of separate manuscript.

### Participants

The primary objective when doing an interpretative phenomenological study is to get a deep understanding from an individual point of view and the focus is less on generalizability. Therefore, homogeneity is preferred for studies employing IPA [23]. Hence, it was ensured that the sample was homogeneous in terms of the phenomenon being studied. Participants had to fulfil the criteria of having had the illness for at least six months duration and were in remission at the time of intake into the study and interview as per the definition given by Mataix-Cols et al. [24]. It is as follows ‘If a structured diagnostic interview is feasible, the person no longer meets diagnostic criteria for OCD for at least one week. If a structured diagnostic interview is not feasible, a score of ≤ 12 on the (C)Y‐BOCS plus Clinical Global Impression ‐ Severity (CGI‐S) rating of 1 (“normal, not at all ill”) or 2 (“borderline mentally ill”), lasting for at least one week.’

Table 2 enumerates the inclusion and exclusion criteria devised for the purpose of the study.

**Table 2:** Inclusion and exclusion criteria

Inclusion criteria
1. DSM-5 diagnosis of obsessive–compulsive disorder 2. Age range: 7–17 years 3. Both male and female patients 4. Duration of illness – 6 months or more 5. Illness in remission 6. Subject should be fluent in English 7. Written Informed Assent from the subject 8. Written Informed Consent from parent or guardian
Exclusion criteria
1. Neurodevelopmental disorders – Intellectual Developmental Disorder (IDD), Autism Spectrum Disorder (ASD) and Attention Deficit Hyperactivity Disorder (ADHD) 2. Presence of psychosis 3. Progressive neurological disorders 4. H/O significant head injury or organic brain disease or substance dependence 5. Presence of any chronic medical illness

### Sample size

**Fig. 1:**
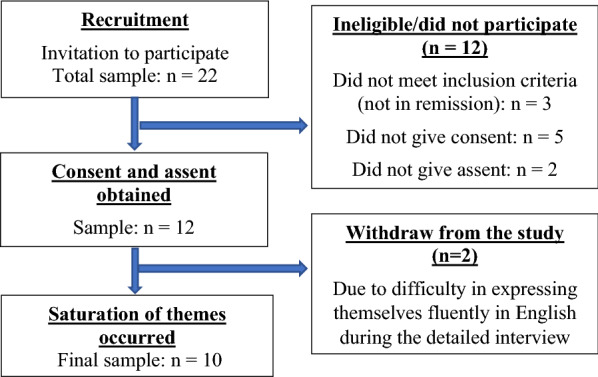
Process of recruitment

Participants were recruited till the saturation of the themes occurred. Fig. 1 depicts the process of recruitment.

### Materials

MINI KID (Mini International Neuropsychiatric Interview for Children and Adolescents) 6.0 [25], Children’s Yale-Brown Obsessive–Compulsive Scale [26] and Clinical Global Impression-Severity Scale [27] were administered at the time of recruitment of subjects into the study. Interviews were conducted using an in-depth semi-structured interview guide and audio-recorded.

### Procedure

All the interviews were undertaken and coded by the first author [LS], a qualified female psychiatrist with substantial experience in collecting and analysing qualitative data, particularly within mental health populations. Children who met the DSM-5 criteria for a clinical diagnosis of OCD were referred to the first author, who then administered the structured diagnostic interview MINI KID (Mini International Neuropsychiatric Interview for Children and Adolescents) 6.0 [25] to confirm the diagnosis and scales—Children’s Yale-Brown Obsessive–Compulsive Scale [26] and Clinical Global Impression-Severity Scale [27] to assess severity at the time of recruitment of subjects into the study. The qualitative data was collected using the in-depth semi-structured interview guide mentioned above. The interview guide was literature guided and validated by a senior researcher of the team with extensive experience in conducting qualitative research. A technique called funnelling was used while constructing the guide. It provides a chance for the participants to express their general views before they are directed to more specifics pertaining to the issues being discussed [28]. Developing the interview schedule was a reflective process keeping in mind that the questions inquire into other people’s lives and how they may affect them [29]. Prompts were also used to enhance the richness of responses, especially in instances when participants had difficulty understanding the questions or talking at length [28]. The questions were open-ended with no right or wrong answers, instead provided an opportunity for a descriptive process.

The participants were allowed to take the lead and it was ensured that all the specific areas were covered. In addition, they were also provided space to voice any other ideas that they felt were relevant. The interviews were audio-recorded to avoid loss of data and recall bias, and, to get data exactly as narrated by the participants. The use of audio recordings helped in understanding the responses better as they contained researcher’s responses and also made it possible to pause it when needed [30].

### Analytic approach

The research question of this study required participants to reflect and talk about their lived experiences and therefore called for a qualitative research methodology and analysis. The study sought to explore participants’ sense-making of their world, of their thoughts, feelings and perceptions through interpretative enquiry. Hence, Interpretative Phenomenological Analysis (IPA) was the chosen method of analysis as it is deals with exploring and understanding the lived experience of a specified phenomenon [31].

### Analysis

IPA is rooted in the philosophy of phenomenology as developed by Husserl and refined by Heidegger. Hermeneutics and idiography are two other elements that form a strong theoretical foundation of IPA. Hermeneutics deals with the interpretation of a subject’s personal world and idiography refers to an in-depth analysis exploring the individual perspectives of participants in their unique contexts [23]. In fact, IPA applies double hermeneutics or a dual interpretation process where the researcher seeks to make meaning out of the meaning-making of others [16]. It views research as a dynamic process involving both researcher and participants.

### Validation of analysis and model

The findings were confirmed in discussion with the  guide and the sub-themes and overarching main themes were corroborated by an independent researcher—a process called investigator triangulation [32]. Member check was done to enhance the rigour of the study. The themes and sub-themes upon which there was agreement were included in the final report and where consensus could not be reached were discarded. All the participants  agreed on the model presented. The final model was then presented to colleagues and members of the team, thus completing peer validation of the model.

### Ethical considerations

Approval to conduct the study was obtained from the Institutional Ethics Committee of the National Institute of Mental Health and Neurosciences (NIMHANS). The children and their parents were provided with written and verbal explanations of the purpose and procedures of the study. Written informed assent and consent were taken from all the participants and their parents respectively. Anonymity and confidentiality were maintained.

## Results

### Clinicodemographic data

All participants were aged from 10 to 17 years of age. The sample constituted of four girls and six boys. All were going to regular school except two (participants 2 and 10) who had dropped out of school due to impaired academic functioning. Participant 2 was training in pre-vocational skills and participant 10 was pursuing studies through open schooling. Age at onset ranged from 9.5 to 13 years (mean-11 years, SD-1.2). Age at diagnosis ranged from 10 to 14.5 years (mean-12.4 years, SD-1.9). The total duration of illness ranged from 10 months to 4 years 6 months (mean-30 months, SD-16). Duration of remission ranged from 12 to 28 weeks (mean-19.7 weeks, SD-6.5). The scores at the time of recruitment were between 0 to 7 (mean-2.7, SD-2.2) indicating subclinical illness in all children [26]. On CGI-Severity rating, seven children scored one indicating ‘normal or not at all ill’ and three children scored two suggesting ‘borderline mentally ill [27].

The IPA analysis done as described in the methodology section yielded five major themes. Each theme and sub-themes are presented below in Table 3. Each theme and sub-theme was validated with at least three significant statements as per the recommended standards for IPA and all the statements were interpreted. However, only one example per sub-theme is presented in Table 4.

**Table 3:** Major themes and sub-themes

Major Theme	Sub-themes
Illness perception changes over time	Confusion, fear and helplessness
	Grief and acceptance
	Clarity sinks in and battle ensues
	Sense of control and hope
Disclosure on a spectrum	No felt need to disclose due to internal barriers
	Felt need but no space to disclose
	Expressed need and compressed grief in family members
	Seek help but face therapist-related barriers
Cascading effects of OCD	Disruptions in sense of self
	Failure to fulfil role functions
	Victim of bullying and social misperceptions
Treatment infuses hope and helps	Initial refusal to seek treatment
	Useful beyond illness
	It is a personal process
Navigating through OCD	Internal battles and chaos
	Wishful thinking
	Wise in retrospect
	Calm after the storm

**Table 4:** Excerpts from transcripts

Sl. no.	Sub-theme	Excerpts from transcript
1	Confusion, fear and helplessness	*“When it started, I was just scared. Very scared gripped in fear as thoughts were very unpleasant. I couldn’t talk about it to anyone […]. In the initial days of being told about my problem, I did not understand. I thought it will not go at all. It will be with me forever. I was afraid. I had a lot of questions about it, but I couldn’t find answers.” (P5, 14 yr old boy)*
2	Grief and acceptance	*“I felt upset and sad ‘Why did I get this type of a disease? Why me? I have unwanted thoughts regularly that make me upset and sad…why can’t I control them?’ I felt sad that whatever the doctors are saying is correct. It took time but I realized it is something I can work on and I can get better.” (P9, 11 yr old boy)*
3	Clarity sinks in and battle ensues	*“After initial sessions, I got clarity that it was something I was suffering from. Although initially it seemed complex and I thought it would be very hard to come out of. [..] I learnt ways to handle it and kept trying. Gradually it became easier.. [....] I worked on it and felt better. So, that motivated me and gave me the courage to continue therapy and work on improving myself.” (P3, 11 yr old boy)*
4	Sense of control and hope	*“Over a period of time, I gained confidence. I felt I could control it” (P8, 12 yr old boy)*
5	No felt need to disclose due to internal barriers	*“I didn’t have any guilt but like I said I just thought I can do it myself. I can handle the situation myself. Every time I used to feel—‘I can do it myself, I don’t need anyone’s help’. (P6, 12 yr old girl)*
6	Felt need but no space to disclose	*“I wanted to talk but I was worried what my parents would think about me if I talked to them about my problem. So, initially I held back from talking about it.” (P10, 17 yr old girl)*
7	Expressed need and compressed grief in family members	*“First time I talked to my father about what was happening to me. He told that I should control it and do meditation […]. I think he was not aware that such a problem could exist or what I don’t know. […] but he has been quite disturbed ever since he started taking me to doctor. As a parent, he would be sad I guess. But he makes sure we go to the doctor.” (P8, 12 yr old boy)*
8	Seek help but face therapist-related barriers	*“I must have seen at least 10 doctors.[....] I used to tell them that I was scratching my hands and that was my problem. […] Actually, when anyone touched me, I would brush over that area but I didn’t talk about my thoughts. I was not asked, and I didn’t talk. [....] simply we were going to different doctors and they were prescribing creams for application.” (P5, 14 yr old boy)*
9	Disruptions in sense of self	*“I was feeling sick! […] I was totally off my character when I was suffering from OCD [..] I used to be like’when will the school be over? What time is it now? How much more time to go? At what time I will go home?’ I didn’t have any interest in attending classes or even studying at home. That was so not me. [....] I am quite social, I want people around me to be happy. I often crack jokes. But in that period I went totally silent. I was totally like a statue, I stayed away from people, alone and everyone around were like – ‘what happened to you?’.” (P3, 11 yr old boy)*
10	Failure to fulfil role functions	*“I had lot of compulsions. [..] my studies started becoming worse. I was very slow, I was very slow in doing things. […] my parents also started giving me bath because, I was very slow and it would delay everything.” (P1, 10 yr old boy)*
11	Victim of bullying and social misperceptions	*“My elder sister once shouted at me for removing all petals of a flower. I did so in an attempt to make it look proper, because I felt something was not okay about it. I feel quite bad about it. Once I went out with friends to eat ice-cream and I felt the ice-cream cone was not in a proper manner. […] the ice-cream cone was not really round in shape so I just asked the shopkeeper to change it. My friends said, ‘what are you doing, we are feeling embarrassed, we are their regular customer, what they will think?. This is a very bad thing that I embarrassed my friends!” (P7, 16 yr old girl)*
12	Initial refusal to seek treatment	*“I did not want to go to a doctor. Not for this problem. My mother kept telling like me ‘You should go. It won’t be embarrassing, they will help you get better’. I still didn’t want to go, but finally I was convinced to go. I was scared what the doctors will think if I talked about what was happening to me because it was really disgusting thoughts, not nice things. So, I felt shy to talk about it” (P1, 10 yr old boy)*
13	Useful beyond illness	*“This phase of life helped me in a way to connect with myself better than being controlled by some part of the brain. [..] I was lazy so I would procrastinate and I was just too scared to try out anything else.. overcoming OCD has definitely helped in developing myself and coming out of other things! Before I wouldn’t be up to trying new things. So, once I agreed to taking treatment for this—I started being open to trying more things.” (P4, 15 yr old girl)*
14	It is a personal process	*“I told my parents not to tell my relatives or friends. I don’t want anyone to know about my disease.” (P9, 11 yr old boy)*
15	Internal battles and chaos	*“So, initially it was difficult, very difficult. There was a lot of confusion and uncertainty and also dismay.[…] at first I was avoiding people. Because when I interacted with them, I had thoughts that were distressing. Later, I was taught to face the situation and stay calm. I used to read too. Reading books helped me relax.” (P3, 11 yr old boy)*
16	Wishful thinking	*“Sometimes, I have a thought—‘if I didn’t have OCD just like my friends, then I would have also – this 2.5yrs of time, I could have had a nice time and spent it more nicely and productively, like they have’. In the sense, they have not wasted their time in any of this stuff.” (P6, 12 yr old girl)*
17	Wise in retrospect	*“After 2-3 months of my admission in the hospital, I was thinking, ‘See what all things happened to me. If I had told in the very beginning itself, 1.5yrs ago when OCD just started. They would have given me tablets and problem could have been solved. I wouldn’t have had so much difficulty.” (P2, 15 yr old boy)*
18	Calm after the storm	*“Tackling OCD was toughest thing I had to do. I learnt that life is not easy. OCD will affect my journey from here on too. I am sure about it [..]. but still life will be okay, it’ll be nice” (P9*, *11 yr old boy)*

**Fig. 2:**
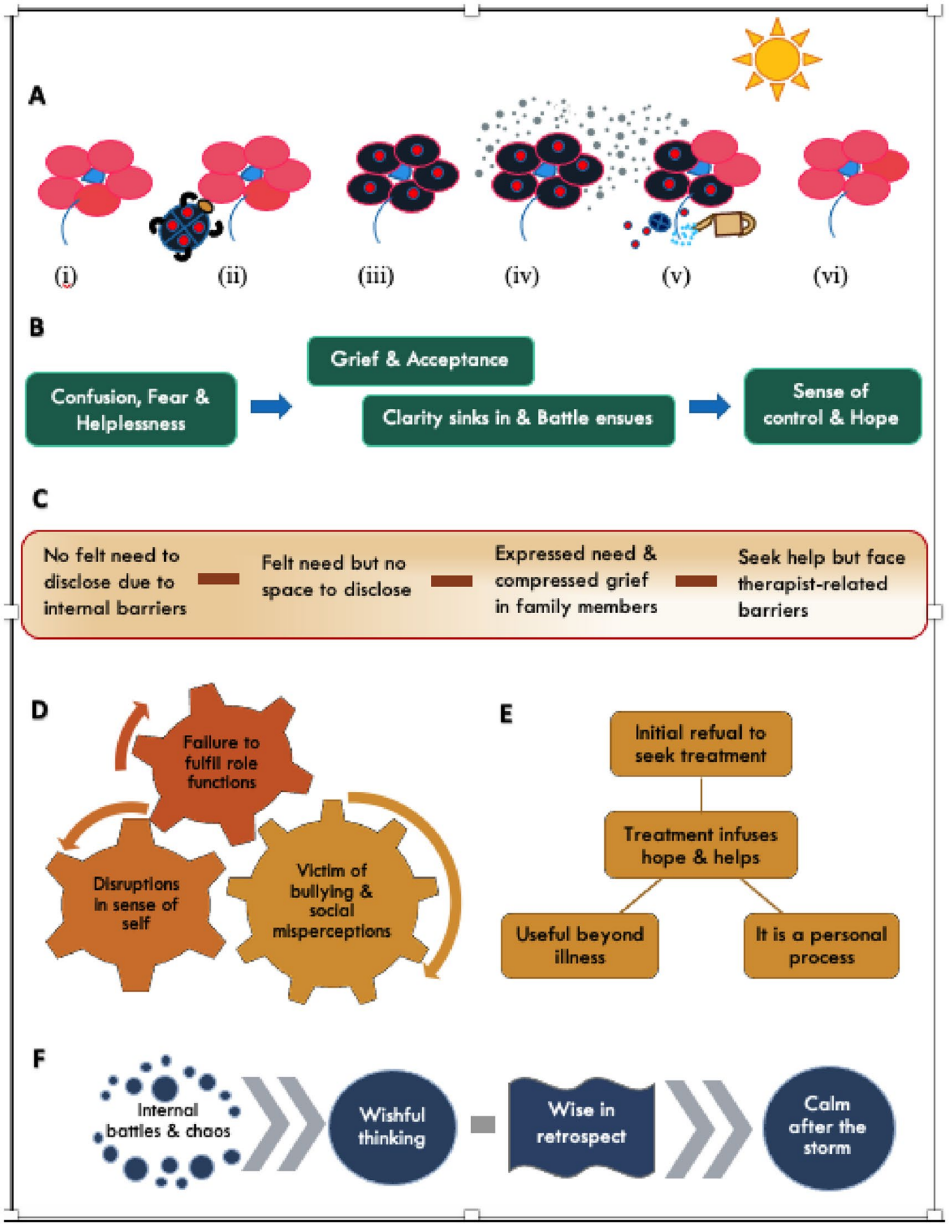
A conceptual model of lived experiences of children with OCD

A summary of these themes is presented as a ‘Conceptual model of lived experiences of the phenomenon of OCD’. The essence of this model is to show the inter-relationship between themes and provide a comprehensive understanding of the phenomenon of OCD as perceived by children (Fig. 2). In addition, there is a pictorial representation of the phenomenon of OCD using analogies of a child’s psyche as a flower and OCD as a bug. So, it has six parts as enumerated and described below.

A. *Phenomenon of OCD:* As mentioned above this is explained by using analogies. A child’s mind is as tender as the petals of a flower [33]. When it is infested by the OCD bug, it is scarred. It also emanates toxins contaminating the surroundings. It is important to recognize the problem and provide a suitable environment for the flower to bloom. While scarring symbolizes the impact of OCD, toxins contaminating surroundings signify the negative impact of OCD on family and a suitable environment here is the right treatment.
B. *Evolution of illness perception:* Illness perception changes over time from initial confusion, fear and a feeling of helplessness to grief and acceptance. As clarity about the condition sets in, a battle with OCD ensues. Later, as one experiences initial successes, a sense of accomplishment and feeling of empowerment emerge. This strengthens determination and gives hope to the individual in their fight with OCD.
C. *Spectrum of disclosure:* Disclosure lies on a spectrum. On one end, there are children who do not feel the need to disclose due to internal barriers such as no felt need for help or lack of insight or awareness. There are others who recognize the need to reveal but do not find the space safe enough to disclose. Some others express, following which the family members go through denial and or ambivalence before coming to acceptance. And at the other end are those who seek help actively but face therapist-related barriers to recognition of the problem.
D. *Cascading effects of OCD:* OCD leads to a chain of events that eventually disrupt one’s role functioning and or cause disruptions in self. It also puts the individual at higher risk of being a victim of bullying as people tend to misinterpret behaviours related to OCD. These sequelae can be interrelated.
E. *Treatment helps as the ‘hub’:* The central theme about treatment is that it helps and this forms the hub of the perception of treatment processes. Although there is initial reluctance to seek treatment, children not only perceive therapy as helpful but helpful beyond illness. However, this process is kept as a personal affair.
F. *Journey through OCD:* It is not a planned journey but a forced one that takes a person by storm. So going through it, children experience a lot of internal battles and chaos within and outside. They face “how I wish I didn’t have it” and “if only I were” moments, in addition to being wise in the retrospect like – “could I have averted this by doing something differently”. Eventually, the storm settles as the individual gets control over the situation and things improve.

## Discussion

Most of the qualitative research on OCD has been done in adult participants. It is focused on exploring subjective experiences [13, 14, 34–42], reassurance seeking [43, 44], enablers and barriers [45], stigma and labeling [46], family members perceptions [47], impact on partner relationships [48] and user perspectives on interventions [49–54]. The sample size in these studies varied depending on the methodology that varied from case study to ethnographic approaches. While Lemelson noted that culture strongly influenced symptomatic expression [36], Olson, Vera & Perez illustrated cultural and ethnic connectedness among their adult participants in their qualitative research [39].

### Comparing study findings with relevant literature

The major themes derived in this study are compared with relevant studies on lived experiences in this field. Table 5 summarizes the methods employed and the key findings of these studies.

**Table 5:** Major studies with which current study findings have been compared

Authors	Phenomenon	Age group	Sample	Method	Key findings
1. Bhattacharya & Singh, 2015 (India)	Subjective experiences	18–25 years	n = 5	Case study approach & thematic content analysis	Three major categories: ‘Connection vs. Disconnection,’ ‘Feeling of Guilt,’ and ‘Authenticity’ [13]
2. Brooks, 2011 (USA)	Lived experience	> 35 years	n = 1	Auto-ethnography	Need for individuals to steer themselves among and between ‘appropriate’ performance and secret rituals; emphasis on ‘importance of image management’ [14]
3. Keyes, Nolte and Williams, 2018 (UK)	Lived experiences	13–18 years (mean -15 years 7 months)	n = 10	Thematic analysis	Four themes: Traumatic and stressful life events; responses to signs of OCD; battle of living with OCD and ambivalent relationship to help; need to address stigma and sense of shame [22]
4. Kohler, Coetzee and Lochner, 2018 (South Africa)	Subjective experiences	18 years or older (mean -45.65 years)	n = 20	Thematic analysis	Three core themes identified—realisation of OCD; disruptions to daily life; and managing the disruptions to daily life [35]
5. Murphy & Perera-Delcourt, 2014 (UK)	Lived experiences	22–53 years	n = 9	IPA	Two major themes: ‘having OCD’ & ‘impact of therapy’ [38]
6. Olson, Vera & Perez, 2007 (Hawaii)	Lived experiences	30–62 years	n = 10	Data analysis by Consensual Qualitative Research	Main themes—Symptoms and meaning, care and treatment, coping and independent living, connectedness [39]
7. Pedley et al., 2019 (UK)	Illness perceptions	16 years or older (2 were between 16–24 years and 14 subjects were above 24 years)	n = 16	Thematic analysis	Recognition of symptoms affected by failure to interpret experiences as ‘symptoms’. Participants tried to decrease its consequences by concealing symptoms [41]
8. Robinson, Rose and Salkovskis, 2017 (UK)	Enablers and barriers to seeking treatment	21–57 years (mean -36 years)	n = 17	Thematic analysis	Barriers -stigma; internal factors (not knowing the problem); treatment-related factors/general practitioner-related factors (GP); & fear of criminalisation. Positive enablers -support to seek help; information regarding OCD in media; confidence in GP. Negative enablers were crisis; feeling driven to seek treatment due to nature of content (thoughts) [45]

#### Theme 1—Illness perception changes over time

The sub-theme of ‘confusion, fear and helplessness’ under this overarching theme corresponds to the sub-themes ‘lack of understanding of their behaviour’ and ‘I thought I was going crazy’ of the major theme of ‘responses to signs of OCD’ reported in a study by Keyes et al. [22]. ‘Recognising something’s wrong’ and ‘coming to terms with OCD’ are two sub-ordinate themes under the major theme of ‘realisation of OCD’ elucidated by Kohler, Coetzee and Lochner [35]. These correspond to the sub-themes of ‘clarity sinks in and battle ensues’ and ‘grief and acceptance’ of the current study. It is important to note that they have not been described in relation to time in the previous study by Kohler, Coetzee and Lochner [35].

Although Murphy and Perera-Delcourt describe ‘having obsessive–compulsive disorder’ as a super-ordinate theme, the concept of evolving over time is not reflected in their description of the minor themes of ‘wanting to be normal and fit in’, ‘failing at life’ and ‘loving and hating OCD’ [38]. Pedley et al. enumerated dimensions of illness perception viz. identity, cause, consequences, timeline, emotional representation, personal control/treatment control and coherence using the Common-Sense Model (CSM), however, these dimensions do not follow a timeline [41]. Olson, Vera and Perez noted that participants tried to make sense of their symptoms both clinically and personally and that symptoms change over time [39].

#### Theme 2—Disclosure on a spectrum

The sub-ordinate theme under this major theme ‘no felt need to disclose due to internal barriers’ describes lack of awareness and lack of insight as coming in way to recognize and talk about the problem. ‘Not wanting to tell people’ and ‘not wanting to tell the doctor’ due to ‘stigma’ as noted by Robinson, Rose and Salkovskis can be compared to this minor theme [45]. Recognition of symptoms was hampered by a failure to interpret experiences as ‘symptoms’ as noted by Pedley et al. However, in that study, the individuals interpreted symptoms as a personality quirk, or as evidence that they had become deviant [41]. In an autoethnographic account, Brooks alludes to secret rituals done in an attempt to maintain a social image [14].

#### Theme 3—Cascading effects of OCD

This major theme has a sub-theme called ‘victim of bullying and social misperceptions’. Bhattacharya and Singh give a detailed account of their participants’ difficulties in sharing experiences of OCD due to social misperceptions as ‘connection vs. disconnection’, which also overlaps with the subtheme of ‘felt need but no space to disclose’ under theme 2 of the current study [13]. ‘Bullying and friendlessness’ under the major theme of ‘traumatic and stressful life events’ described by Keyes et al. reflects traumatic life events occurring in the months immediately preceding the onset of OCD [22]. However, the sub-theme ‘victim of bullying and social misperceptions’ under ‘cascading effects of OCD’ refers to bullying occurring in the aftermath of OCD. Kohler, Coetzee and Lochner described an overarching theme ‘disruptions to daily life’ that encompassed sub-ordinate themes of ‘disruptions in sleep and rest’, ‘disruptions to leisure activities and hobbies’ and ‘disruptions to productivity [35]. The present study had ‘disruptions in sense of self’ as one of the sub-themes. While Bhattacharya and Singh described ‘feeling different from others’ and the ‘loss of an authentic self’ [13], Brooks elaborated on the disruptions to social life and the impact of OCD on public and private identities leading to significant suffering [14].

#### Theme 4—Treatment infuses hope and helps

This major theme corresponds to the sub-ordinate theme of ‘wanting therapy’ under ‘impact of therapy’ as elucidated by Murphy and Perera-Delcourt. Moreover, subtheme ‘useful beyond illness’ corresponds to the subtheme of ‘a better self’ [38]. It is interesting to note that one of the other sub-themes ‘initial refusal to seek treatment’ is similar to the super-ordinate theme ‘ambivalent relationship to help’ identified by Keyes et al. [22].

#### Theme 5—Navigating through OCD

One of the sub-themes of this major theme is ‘internal battles and chaos’ that occurs in the initial phase of the illness. Brooks illustrated a battle against OCD in her personal account of steering herself among and between “appropriate” performance and secret rituals [14]. Keyes et al., also identified ‘the battle of living with OCD’ illustrating the struggle of adolescents to cope with internal experiences related to their difficulties [22].

### Clinical implications of themes derived

Despite the developmental differences, there is an overlap in the lived experiences of children and adults having OCD. It is important to be aware of how the perception evolves over time, be it of the illness or treatment processes.

- After coming to acceptance, children develop hope while battling OCD. Therapists must be aware of it and not be too aggressive but be supportive during the initial phase.
- Children found therapy to be helpful beyond illness in terms of inculcating self-discipline. It is likely to positively impact other spheres of the child’s life such as academics and interpersonal relationships and result in more long-term benefits.

### Strengths and limitations

To the best of our knowledge this is the first qualitative study in children and adolescents with OCD done using interpretative phenomenological analysis, which is the choice of analysis for exploring lived experiences. The youngest subject in this study was ten years old. To our knowledge, this study reports the youngest child to narrate lived experiences of OCD. The average duration of interviews was 1 h and 49 min, which meant a lot of data for comprehensive and a holistic understanding of each subject. As it is a qualitative study, the data were intended to provide in‐depth insights into subjective experiences, rather than to be generalizable. The research team chose to study only English speaking participants to ensure uniformity and sociodemographic homogeneity (lifestyle, social scrutiny and support) of the sample studied. We ensured ‘clinical homogeneity’ by having subjects who had the illness for at least six months duration and were in remission at the time of intake into the study. However, the researchers wanted to explore the experiences of children who suffered varying intensity of the disease, hence the sample was heterogeneous in terms of the severity of illness endured. The transcribed data was meticulously analysed to derive the results. While all subjects were interviewed at length and a lot of data was collected, only the overlapping themes have been elucidated in detail. The subtleties of individual experiences were deliberately missed in the process of drawing conclusions from a large amount of data. It is noteworthy that despite the developmental differences due to the broad age group of the sample studied (10–17 years), the themes of lived experiences of OCD that emerged were the same in all the subjects. After much deliberation, it was decided to include subjects in remission from illness so as to get a sense of the experience of the ‘whole journey’ through the illness and the treatment processes during different phases. While this could be a strength of the study, it is not the same as getting narratives from those who are acutely ill. There could have been a recall bias. But, it is unlikely that the most prominent and difficult experiences would not have been recounted by the subject. There is a possibility that subtle features would have been missed while narrating their experiences.

### Recommendations for future research

This study suggests that major themes of illness perception and treatment processes evolve over time. Longitudinal follow-up studies would be required to establish this. Qualitative research can be carried out with a focus on the specific issue related to disclosure, i.e. ‘therapist-related barriers’ as there is scope to understand the processes better and improve them accordingly to facilitate disclosure and thereby identify and address the phenomenon of OCD early. As there is a lack of research on the impact of OCD on the developing identities of children, it would be worthwhile studying this aspect too by conducting qualitative research. Children talked about their difficulties in the school context and often being misunderstood by friends and or teachers. Moreover, children spend a substantial amount of time in school. Therefore, it would be worthwhile studying the student–teacher dyads as it will not only provide an opportunity to understand teachers’ perspectives but also inform policies related to school.

## Data Availability

Transcripts will not be shared because study participants did not give their approval in the informed consent.

## Abbreviations

CBT: Cognitive behaviour therapy
IPA: Interpretative phenomenological analysis
MINI KID: Mini International Neuropsychiatric Interview for Children and Adolescents
NIMHANS: National Institute of Mental Health and Neurosciences
OCD: Obsessive–compulsive disorder
SD: Standard deviation
